# Reliable Information from Health Professionals Encourages Urban Japanese Mothers’ Continued Participation in Health Checkups

**DOI:** 10.3390/healthcare10081523

**Published:** 2022-08-12

**Authors:** Rumi Tsukinoki, Yoshitaka Murakami, Haruhiko Imamura, Tomonori Okamura

**Affiliations:** 1 Department of Public Health Nursing, Tokyo Medical and Dental University, Tokyo 113-8519, Japan; 2 Department of Medical Statistics, School of Medicine, Toho University, Tokyo 143-8540, Japan; 3 Graduate School of Health and Nutrition Sciences, The University of Nagano, Nagano 380-0845, Japan; 4 Department of Preventive Medicine and Public Health, Keio University, Tokyo 160-8582, Japan

**Keywords:** health checkups, health information, mothers’ health, health professional

## Abstract

We examined mothers’ health information sources and their relationships with continued participation in health checkups among urban Japanese mothers. Participants were 152 mothers below 40 years old with one or more children under 12 years old. We collected data at a children’s festival in Tokyo in 2019. A self-administered questionnaire was used to collect information regarding health checkups, trusted sources of information regarding mother’s health, and anthropological variables. Continued participation in health checkups was defined as participating in health checkups almost every year during the past five years. Logistic regression analysis was used to adjust for health insurance, mothers’ age, number of children, and current medical history. the sources of mothers’ health information trusted by over 20% of mothers in the two groups were “family”, “friends”, “Web/SNS”, and “healthcare professionals.” However, continued participation in health checkups was significantly associated with only the source of health information from “healthcare professionals” (odds ratio: 2.8 [95% confidence interval: 1.26–6.31], *p* = 0.01). These findings suggest that reliable information from health professionals encourages urban Japanese mothers’ continued participation in health checkups among Japanese mothers under 40 years old who have children under 12 years of age.

## 1. Introduction

Many women complain of physical and mental problems due to hormonal changes and childcare-related fatigue postpartum, including postpartum depression, weight retention, gestational hypertension, diabetes, and metabolic risk [1]. This suggests the importance of following up with health examination or management of mothers for several years after childbirth. Mothers with gestational diabetes or gestational hypertension are at an increased risk of type 2 diabetes and hypertension [2]. Moreover, in women, hypertension, diabetes, and metabolic syndrome increase sharply after 40 years old, especially after menopause [3,4,5]. However, the participation rate in health checkups is relatively low among women who are homemakers or part-time workers, particularly those who are busy with childcare or nursing in Japan [6,7]. Changing social and environmental factors is necessary to increase this participation rate for mothers over 40 years old and make it a habit for mothers under 40 years of age to receive health checkups. 

Appropriate health information is important for improving participation in health checkups in mothers under 40 years old. Social networks, health consciousness, and health knowledge can encourage participation in health examinations [6,8]. We examined the relationships between the sources of health information regarding women’s health and continued participation in health checkups among urban Japanese mothers under 40 years old.

## 2. Materials and Methods

We collected data at a babies’ and children’s festival in the Shinagawa ward in Tokyo in 2019. Mothers below 40 years old with one or more children under 12 years old were included in our study. A total of 155 mothers participated in this survey. In the participants, the important information of three mothers was missing and thus excluded. Finally, 152 participants were included in the analysis. The survey was a self-administered questionnaire. We asked mothers about their age, the number and age of their children, employment status, health insurance, participation in health checkups, pregnancy status, health conditions, and perceived reliability of sources of information regarding mothers’ health, childcare, and children’s education. The mother’s weight and height were asked by the self-reported survey. Blood pressure was assessed using a cuff-oscillometric device (HEM-7080IC; Omron Healthcare Co., Ltd., Kyoto, Japan). Blood pressure was recorded in a sitting position after at least 3 min of rest. 

Continued participation in health checkups was defined as participating in health checkups almost every year (4–5 times) during the past five years with a self-administered questionnaire.

The relationship between continued participation in checkups and trusted sources of information regarding women’s health was examined using logistic regression. The relationship between continued participation in checkups and trusted sources of information regarding women’s health was examined using logistic regression. In the logistic analysis, the dependent variable was continued participation in health checkups; the independent variable was “friends”, “family”, “healthcare professionals”, and “Web/SNS” as reliable sources of information for mothers’ health behavior. These four sources of maternal health information entered into the model were those selected by ≥20% of both groups in multiple-choice questions about “sources of information that they trust and refer to regarding their health behavior.” The logistic analysis was also adjusted for the type of mother’s age, the number of children, the health insurance (insured or dependent), and the mother’s current health problems (yes/no).

All analyses were performed using STATA16. Statistical significance was set at a two-tailed *p*-value of less than 0.05. This study was approved by the Institutional Review Board of the Japanese Red Cross College of Nursing (2019-062). All participants provided both written and oral consent to participate.

## 3. Results

In Table 1, The mean age of mothers was 32.0 (standard deviation: 5.3) years in the no participation in health checkups group and 33.2 (3.2) years in the continued participation in health checkups group. The percentage of mothers with one child was 71.7% in the no continued participation in health checkups group and 77.2% in the continued participation in the health checkups group. The percentage of mothers who were insured (employees) was 27.2%, different from 50.0% in the no continued participation in health checkups group and 77.2% in the continued participation in the health checkups group.

Table 2 shows reliable sources of health information for mothers’ behavior by those who participated or did not participate in health checkups. The sources of mothers’ health information trusted by over 20% of mothers in each group were “family”, “friends”, “Web/SNS”, and “healthcare professionals”.

Table 3 shows the association between reliable sources of health information regarding mothers’ health behavior and continued participation in health checkups. Continued participation in health checkups was significantly associated with only the source of health information from “healthcare professionals” (odds ratio: 2.8 (95% confidence interval: 1.26–6.31), *p* = 0.01).

## 4. Discussion

The current findings revealed that, among Japanese mothers, those who participated in health checkups were significantly associated with trusting information from healthcare professionals compared with those who did not participate in health checkups. Previous studies reported that many mothers consider maternal health information from healthcare professionals to be reliable [9,10,11]. However, the current study is the first to examine the relationship between differences in continued participation in health checkups and the perceived reliability of sources of information regarding mothers’ health among urban Japanese mothers under 40 years old. In a previous study, emphasizing the maternal health literacy of mothers by healthcare professionals was also strongly recommended [11]. Another study showed that trust in physicians exceeded that of any other information channel for health or medical information, and the trend was strongest for respondents who were young, educated, and women [12]. Health knowledge encouraged participation in health examinations [8]. Furthermore, making the health checkup a habit may promote communication with healthcare professionals and trust in information from the healthcare professionals. These findings suggest that the health information from healthcare professionals may be essential for promoting continued participation in health examinations among Japanese mothers under 40 years.

The current results also revealed that health information from “healthcare professionals” also suggests encouraging healthy behavior in Japanese mothers under 40 years. Few papers showed the relationship between health behaviors and information sources among Japanese mothers. Yamamoto et al. showed that pregnant Japanese women who obtained information from medical staff were prevalent among folic acid takers among pregnant Japanese women with a mean age of 33 years in Osaka [13]. Using an Internet marketing survey of pregnant Japanese women with a mean age of 32 years, Shono et al. showed that the main source of information recommending maternal influenza vaccination was an obstetrician, followed by nurses/midwives among vaccinated takers [14]. Globally, some studies reported similar findings that healthcare professionals are the most common information sources used by women with children. A previous study of pregnant women with a mean age of 30 years in the Netherlands reported that pregnant women mainly obtained health information from midwives [9]. In a prospective cohort study of mothers with a mean age of 30 years in Adelaide, women with their first child commonly got health information from their parents and healthcare practitioners during pregnancy [10]. When children are school-aged, 78% of mothers reported the most trust in information from healthcare professionals [10]. In a study of mothers with a median of 28 (14–46) years old in Laos [11], the main source of mother and child information was healthcare professionals. Even though information sources such as digital are increasing and varied, health professionals’ sources are still the most commonly trustworthy and helpful among pregnant women and mothers. Therefore, healthcare providers should understand carefully what information mothers seek for health behaviors and continued participation in health checkups.

The current study is the first to examine the relationship between differences in continued participation in health checkups and the perceived reliability of sources of information regarding mothers’ health among urban Japanese mothers under 40 years old. This study shows that mothers’ health information provided by health professionals is essential for promoting continued participation in health checkups. In a previous study, emphasizing the maternal health literacy of mothers by health professionals was strongly recommended. Strategies and activities to strengthen health workers’ communication skills could help to improve mothers’ health literacy [11]. Health information was also a positive factor for continued participation in health checkups in the current study. Thus, providing health information by health professionals may effectively promote continued participation in a health examination.

This study involved several potential limitations. First, the causal relationships remain unclear because this was a cross-sectional study. Second, the generalization of our study findings should be cautioned. All participants were mothers below 40 years old with one or more children under 12 years old in Tokyo. However, this study’s main sources of mothers’ information on health behavior exhibited the same trends as those reported in previous studies in other countries [9,10,11]. This finding may be speculated about the ability to generalize. Further investigation will be required to examine this relationship among women in other communities in Japan. Third, the data were collected in 2019 before the COVID-19 pandemic. The COVID-19 pandemic resulted in numerous changes in the delivery of healthcare services, including cancer screening and management of chronic disease [15,16,17]. Although face-to-face contact between healthcare professionals and patients has decreased, healthcare professionals have adapted to new ways of virtual healthcare and digital technologies [16,17]. Women may have easier access to health information in a variety of ways. Further study will be required to examine this relationship among women after the COVID-19 pandemic.

## 5. Conclusions

These findings suggest that reliable information from health professionals encourages urban Japanese mothers’ continued participation in health checkups among Japanese mothers under 40 years old and have children under 12 years of age. Continuous provision of information regarding mothers’ health by healthcare professionals may make it a habit for mothers under 40 years old to receive health checkups.

## Figures and Tables

**Table 1:** Participants’ characteristics (N = 152).

	No Continued Participation in Health Checkups N = 60		Continued Participation in Health Checkups N = 92	
	Means	(SD)	Means	(SD)
Age (years)	32.0	(5.3)	33.2	(3.2)
Weight (kg) *	51.7	(9.0)	51.9	(7.1)
Systolic blood pressure (mmHg) **	110.5	(13.4)	107.9	(11.4)
Diastolic blood pressure (mmHg) **	77.4	(11.4)	75.2	(11.0)
	N	(%)	N	(%)
Numbers of children				
1	43	(71.7)	71	(77.2)
2	15	(25.0)	18	(19.6)
3, 4	2	(3.3)	3	(3.3)
Type of health insurance				
insured (employees)	30	(50.0)	71	(77.2)
dependent (no employees or part-time)	30	(50.0)	21	(22.8)
Current health problems				
Yes	22	(36.7)	39	(42.4)

Continued participation in health checkups was defined as participation in health checkups almost every year (4–5 times) during the past five years: mothers who do not undergo health checkups = 0 (N = 60); mothers who undergo health checkups = 1 (N = 92). (Excluding cancer screening): SD: standard deviation. * Weight: No continued participation in health checkups N = 58; Continued participation in health checkups N = 88. ** Blood pressure: No continued participation in health checkups N = 56; Continued participation in health checkups N = 90. Current health problems: hypertension, diabetes, hyperglycemia, edema, albuminuria, renal disorder, headache, low back pain, stiff shoulder, and palpitation.

**Table 2:** Reliable sources of health information for mothers’ behavior by those who participated or did not participate in health checkups (N = 152).

	No Continued Participation in Health Checkups (N = 60)		Continued Participation in Health Checkups (N = 92)	
	n	(%)	n	(%)
**Mothers’ health**				
Friends	30	(50.0)	36	(39.1)
Family	33	(55.0)	42	(45.7)
Kindergarten, nursery school, school	4	(6.7)	2	(2.2)
Healthcare professionals	13	(21.7)	38	(41.3)
Web and SNS	26	(43.3)	41	(44.6)
Childcare support activities	9	(15.0)	16	(17.4)
City bulletin	4	(6.7)	14	(15.2)
TV	9	(15.0)	10	(10.9)
**Childcare and children’s education**				
Friend	55	(91.7)	70	(76.1)
Family	26	(43.3)	38	(41.3)
Kindergarten, nursery school, school	12	(20.0)	21	(22.8)
Healthcare professionals	19	(31.7)	38	(41.3)
Web and SNS	33	(55.0)	45	(48.9)
Childcare support activities	21	(35.0)	39	(42.4)
City bulletin	10	(16.7)	20	(21.7)
TV	5	(8.3)	7	(7.6)

Multiple answers were possible. No continued participation in health checkups: mothers who did not undergo health checkups almost every year (4–5 times) for the past five years (excluding cancer screening): Continued participation in health checkups: mothers who underwent health checkups almost every year (4–5 times) for the past five years (excluding cancer screening).

**Table 3:** Association between perceived reliability of sources of information regarding mothers’ health behavior and continued participation in health checkups (N = 152).

Reliable Sources of Information for Mothers’ Health Behavior	No Continued Participation in Health Checkups N = 60 N (%)	Continued Participation in Health Checkups N = 92 N (%)	Odds Ratios	(95% CIs)	*p* Values
Friends	30 (50.5)	36 (39.1)	0.53	(0.25–1.10)	0.09
Family	33 (55.0)	42 (45.7)	0.78	(0.38–1.62)	0.51
Healthcare professionals	13 (21.7)	38 (41.3)	2.82	(1.26–6.31)	0.01
Web/SNS	26 (43.3)	41 (44.6)	1.12	(0.55–2.29)	0.76

The dependent variable is continued participation in health checkups almost every year (4–5 times) during the past five years; the independent variable were “friends”, “family”, “healthcare professionals”, and “Web/SNS” as reliable sources of information for mothers’ health behavior; adjusted for the type of mother’s age, the number of children, health insurance (insured or dependent), and mother’s current health problems (yes/no).

## Data Availability

Not applicable.

## References

[1] Paladine H.L., Blenning C.E., Strangas Y. (2019). Postpartum Care: An Approach to the Fourth Trimester. Am. Fam. Physician.

[2] Institute of Medicine (US) and National Research Council (US) Committee to Reexamine IOM Pregnancy Weight Guidelines (2009). Weight Gain during Pregnancy: Reexamining the Guidelines.

[3] Tabaei B.P., Chamany S., Perlman S., Thorpe L., Bartley K., Wu W.Y. (2019). Heart Age, Cardiovascular Disease Risk, and Disparities by Sex and Race/Ethnicity Among New York City Adults. Public Health Rep..

[4] Reeves A.N., Elliott M.R., Brooks M.M., Karvonen-Gutierrez C.A., Bondarenko I., Hood M.M., Harlow S.D. (2021). Symptom clusters predict risk of metabolic-syndrome and diabetes in midlife: The Study of Women’s Health Across the Nation. Ann. Epidemiol..

[5] Wenger N.K., Arnold A., Bairey Merz C.N., Cooper-DeHoff R.M., Ferdinand K.C., Fleg J.L., Gulati M., Isiadinso I., Itchhaporia D., Light-McGroary K. (2018). Hypertension Across a Woman’s Life Cycle. J. Am. Coll Cardiol..

[6] Imamura H., Kogure M., Kita Y., Nakagawa H., Hozawa A., Okamura T., Murakami Y., Nishi N., Ouda N., Kadota A. (2018). Factors related to participation in health examinations for Japanese National Health Insurance: NIPPON DATA2010. J. Epidemiol..

[7] Tsurugano S., Inoue M., Eiji Y. (2012). Precarious Employment and Health: Analysis of the Comprehensive National Survey in Japan. Ind. Health.

[8] Hui-Ting H., Yu-Ming K., Shiang-Ru W., Chia-Fen W., Chung-Hung T. (2016). Structural Factors Affecting Health Examination Behavioral Intention. Int. J. Environ. Res. Public Health.

[9] Vogels-Broeke M., Daemers D., Budé L., de Vries R., Nieuwenhuijze M. (2022). Sources of information used by women during pregnancy and the perceived quality. BMC Pregnancy Childbirth.

[10] Plutzer K., Keirse M.J. (2012). Effect of motherhood on women’s preferences for sources of health information: A prospective cohort study. J. Community Health.

[11] Phommachanh S., Essink D.R., Wright P.E., Broerse J.E.W., Mayxay M. (2021). Maternal health literacy on mother and child health care: A community cluster survey in two southern provinces in Laos. PLoS ONE.

[12] Hesse B.W., Nelson D.E., Kreps G.L., Croyle R.T., Arora N.K., Rimer B.K., Viswanath K. (2005). Trust and sources of health information: The impact of the Internet and its implications for health care providers: Findings from the first Health Information National Trends Survey. Arch. Intern. Med..

[13] Yamamoto S., Wada Y. (2018). Awareness, use and information sources of folic acid supplementation to prevent neural tube defects in pregnant Japanese women. Public Health Nutr..

[14] Shono A., Hoshi S.L., Kondo M. (2020). Maternal influenza vaccination relates to receiving relevant information among pregnant women in Japan. Hum. Vaccin Immunother..

[15] Schifferdecker K.E., Vaclavik D., Wernli K.J., Buist D.S.M., Kerlikowske K., Sprague B.L., Henderson L.M., Johnson D., Budesky J., Jackson-Nefertiti G. (2021). Women’s considerations and experiences for breast cancer screening and surveillance during the COVID-19 pandemic in the United States: A focus group study. Prev. Med..

[16] Chudasama Y.V., Gillies C.L., Zaccardi F., Coles B., Davies M.J., Seidu S., Khunti K. (2020). Impact of COVID-19 on routine care for chronic diseases: A global survey of views from healthcare professionals. Diabetes Metab. Syndr..

[17] McGrowder D.A., Miller F.G., Vaz K., Anderson Cross M., Anderson-Jackson L., Bryan S., Latore L., Thompson R., Lowe D., McFarlane S.R. (2021). The Utilization and Benefits of Telehealth Services by Health Care Professionals Managing Breast Cancer Patients during the COVID-19 Pandemic. Healthcare.

